# Prevalence of oral mucosa lesions in diabetic patients: a preliminary study

**DOI:** 10.1016/S1808-8694(15)30578-4

**Published:** 2015-10-19

**Authors:** Belmiro Cavalcanti do Egito Vasconcelos, Moacir Novaes, Francisco Aurelio Lucchesi Sandrini, Almir Walter de Albuquerque Maranhão Filho, Leila Santana Coimbra

**Affiliations:** 1 PhD, Associate Professor. Coordinator of Doctorate and Masters in BMF Surgery and Traumatology – University of Pernambuco.; 2 PhD, Associate Professor at University of Pernambuco. Coordinator of the diabetes program at Oswaldo Cruz University Hospital, University of Pernambuco, Recife, Pernambuco.; 3 Master in BMF Surgery and Traumatology - PUC-RS, Doctorate in BMF Surgery and Traumatology - UPE.; 4 Graduation in Dentistry, Dental Surgeon.; 5 Graduation in Dentistry, Dental Surgeon. University of Pernambuco.

**Keywords:** candidiasis, diabetes complications, diabetes mellitus, mouth diseases, tongue varicose veins

## Abstract

**Aim:**

The present study aimed to evaluate the prevalence of superficial lesions in the oral cavity mucosa in diabetic patients.

**Methods:**

The sample was made of 30 patients. To obtain these results we did rigorous clinical and complementary tests.

**Results:**

Of the 30 patients, 9 (30%) were males and 21 (70%) females. Of the studied patients, 40% were below 60 years of age, and 60% were older than 60 years. Thirteen different types of mucosal alterations were diagnosed. Tongue varicose veins (36.6%) and candidiasis (27.02%) were the most prevalent. Such alterations can be associated with the fact that these conditions are commonly found in senile patients and are also associated with prolonged wear of dentures. Xerostomia was diagnosed in only 1 (3.33%) patient, disagreeing with most of the studies observed in the literature.

**Conclusion:**

Most of the diabetic patients presented at least one type of oral mucosa lesion or alteration.

## INTRODUCTION

The World Health Organization (WHO) has considered diabetes mellitus a public health problem since 1975^1^. Therefore, it is necessary that health care professionals become interested on the disease in order to provide an appropriate treatment to these patients in the different fields of knowledge.

Diabetes is a dangerous disease since the patient's and healthcare promoter's negligence may impair the patient's quality of life and even lead the patient to death. Diabetes is a disease in which the insulin's regulatory activity is defective. This can be a result of decreased amount of insulin that should be secreted, total absence of insulin secretion or the production of antibodies against insulin causing its destruction before it can act in the different areas of the body^2^. In the first two cases there is degeneration or inactivation of beta cells of the Langerhans islets which produce insulin. In the last case, the amount of insulin secreted may be normal but it does not reach its destination^2^.

In 1997, the American Association of Diabetes proposed a classification system for diabetes based on its etiology. Therefore, diabetes is currently classified as: Type 1 or juvenile diabetes and Type 2 or acquired diabetes. Type 1 diabetes appears in the first or second decade of life; it is caused by the destruction of pancreatic beta cells, which can be caused by a viral or an autoimmune process leading to a blockade in the production of insulin^3^. On the other hand, type 2 diabetes is the result of an abnormality that can occur both at the molecular level of insulin and at the cellular level of insulin receptors^3^.

In 2005, Rivera et al.^4^ suggested that the appearance of Alzheimer disease may be associated with a new type of diabetes named Type 3 diabetes by the authors. Although the pancreas is the main organ responsible for insulin secretion, the fall of insulin levels in the brain causes the so-called type 3 diabetes. In this study it was found that the brain produces a small amount of insulin and proteins. The fact that there is an appropriate level of insulin as well as the correct activity of the receptors is described as vital for the cell survival in the brain.^4^

It is estimated that there are about 170 million people with diabetes mellitus in the world and approximately 10 million in Brazil. Of those, approximately 50% do not know they have the disease^3^. According to the WHO, about 7% of the world adult population has diabetes. In the state of São Paulo this rate reaches 9.6%^6^.

Paradella, Monteiro da Silva and Arisawa^1^ state that the main symptoms of the patient with diabetes mellitus are polydipsia, polyuria-nycturia, polydipsia associated with xerostomia, polyphagia, vulvar pruritus, rapid weight loss, even with a balanced diet. Visual changes (such as blurred vision), somnolence, pain, cramps, fatigue, tingling and numbness of lower limbs, asthenia, organ deficiency, indisposition to work, discouragement, generalized physical and mental tiredness, ketoacidosis and fruit breath are also observed^1^.

In regards to the specific role of Otorhinolaryngology, Scherer and Lobo^7^ noticed irritative vestibular disorder in patients with type I diabetes. Maia and Campos^8^ state that there is evidence that diabetes mellitus may cause hearing loss.

According to Tommasi^9^, the most common oral manifestations in diabetic patients include xerostomia, burning and eventual erythema, ulcerations, pharyngeal infections caused by Candida albicans, cheilitis, lichen planus, tumefaction of salivary glands, gingival problems, periodontal problems, abscesses and marked loss of alveolar bone, although none of them is a pathognomonic lesion^3^. In the patient with uncontrolled diabetes, a decreased response to infection (bacterial, fungal and viral) is observed, due to the hyperglycemia and ketoacidosis that changes the phagocytosis of macrophages and the chemotaxis of polymorphonuclear neutrophils. The patient with controlled diabetes without vascular disease does not present increased rates of infection since a good control of the disease reduces the likelihood of infection to a minimum, and repair does not seem to be very different from that seen in the non-diabetic patient^9^. In 1993, the WHO included the periodontal disease as a classic complication of diabetes^10^.

The clinical manifestations and the oral symptoms of diabetic patients may vary from a minimum to a more aggressive stage and depend on the type of existing hyperglycemic abnormality, of treatment control and the time elapsed since the diagnosis of the disease^11^.

Diabetes requires a deep knowledge by all healthcare professionals involved in the diagnosis oral lesions since it has several intervening factors in the patient's oral condition. Therefore, it is necessary to know how to correctly diagnose, prescribe and manage the diabetic patient, thus eliminating the risk of complications and at the same time improving the patient's quality of life.

Given the importance of the disease and the need for deeper knowledge on oral abnormalities to which the diabetic patients are subject to, the purpose of this investigation was to study the prevalence of the superficial lesions in the oral mucosa in a group of patients with diabetes mellitus.

## MATERIALS AND METHODS

This is a cross-sectional study, characterized as a case series of observational character. After file consultations, the diabetic patients seen in 2005 were selected. With the identification data, all the patients were invited to a dental visit in the same sector by means of a postal communication. In 2005, a total of 70 patients were seen in the aforementioned service; 30 patients agreed to participate on the study. Smokers, patients who drank alcohol on a regular basis or patients with an immunosuppressant disease associated with diabetes were taken off the study. After physical examination, patients who presented lesions associated with diabetes were instructed and referred to appropriate treatment when necessary.

During the clinical examination, all the data was recorded in a medical card created for the study. The diagnosis of lesions was established by the anamnesis and physical examination, and when necessary, by the incision biopsy and histopathological examination. The analysis and records of lesions we did not take into account caries and periodontal disease.

This study was approved by the Research Ethics Committee of the University of Pernambuco under number 008/06. All the patients were informed about the research character and agreed to participate on the study by signing the free and informed consent form.

## RESULTS

The ages of participants in this research varied between 20 and 79 years old with a mean of 61.53 years, standard deviation of 11.44 years, and coefficient of variation of 18.60%. The median age was 64 years.

Of the 30 patients, 18 (60%) were older than 60 years and 12 patients were up to 60 years old. As to gender, 70% (21) were females and 30% (9) were males.

Most patients (93.3%) had type 2 diabetes. The period between disease onset (diabetes) and the date of physical examination varied between 5 and 45 years, with the intervals 5 to 10 and 11 to 20 the most frequent periods in 44.4% of the sample in each range. This information was not available for 3 patients. Of 30 patients, 27 (90%) were receiving drug treatment and 10% did not use medication either for control of diabetes or other associated disease according to Table 1.

**Table 1:** Distribution of studied individuals according to the variables related to diabetes..

Variable	n	%
• Type of diabetes		
1	2	6,7
2	28	93,3

TOTAL	30	100,0

• Time since the onset of diabetes (years))		
5 a 10	12	44,4
11 a 20	12	44,4
21 a 45	3	11,1

TOTAL(1)	27	100,0

• Regular use of medication		
Yes	27	90,0
No	3	10,0

TOTAL	30	100,0

n - number;

Of the 30 patients, only 1 (3.3%) sporadically drank alcoholic beverages (for more than 5 years). Oral health was fair in 27 patients (90.0%), good in 2 patients (6.7%) and poor in only 1 patient (3.3%).

Table 2 shows the conditions seen in the oral cavities of the individuals studied and their respective sites. This table depicts a total of 37 lesions of 13 different types of oral mucosa abnormalities. The most frequent abnormality was lingual varicosity, with 11 cases (located in the tongue floor), followed by 10 cases of erythematous candidiasis, (7 cases located in the hard palate and 3 cases in the attached gingiva).

**Table 2:** Evaluation of oral cavity conditions and their respective sites.

				Sites				
	1	2	3	4	5	6	7	
Conditions								Number of lesions
Cheilitis	-	-	-	2	-	-	-	2
Traumatic ulcer	1	-	-	-	-	-	1	2
Fissured tongue	-	-	-	-	-	-	1	1
Gingival hyperplasia	1	-	-	-	1	-	-	2
Atrophy of lingual papilla	-	-	-	-	-	-	1	1
Erythematous candidiasis	-	7	-	-	-	3	-	10
Mucocele or ranula	-	-	-	-	1	-	-	1
Xerostomia				No specific site				1
Lingual varicosity	-	-	11	-	-	-	-	11
Racial pigmentation	-	-	-	-	-	1	-	1
Gingivitis	-	-	-	-	-	3	-	3
Petechia	-	-	1	-	-	-	-	1
Hyperkeratosis	-	-	1	-	-	-	-	1

TOTAL	2	7	13	2	2	7	3	37

1 – Right upper posterior alveolar ridge; 2 – Hard palate; 3 – Lingual floor; 4 – Labial angle; 5 – Oral floor; 6 – Attached gingiva; 7 – Dorsum of the tongue;

Of 30 patients, 24 (80.0%) presented at least one lesion or mucosal abnormality and 6 (20.0%) did not present any oral lesion or abnormality.

## DISCUSSION

The study of oral mucosa abnormalities in diabetic patients is important due to the need of greater knowledge about the oral abnormalities in these individuals. The importance increases given the conflicting results in regards to the prevalence of oral abnormalities seen in the literature, in addition to the fact that diabetes is a worldwide health problem. Guggenheimer et al.^12^ reported that this variable prevalence of oral abnormalities may be a reflection of the different physiological behaviors of the two clinical types of diabetes. Other factors that can also be responsible include variations in glucose control, duration of the disease and patient's age.

Diabetes mellitus has a worldwide distribution, occurring in about 1 to 2% of the world population, and it is more prevalent in well fed populations because they have better access to mostly high-calorie foods. The incidence of diabetes is predominantly in adult age, with 85% of the individuals above 40 years old who develop the disease due to poor instructions on health prevention and dietary control. Only 5% of patients present the disease before they are 20 years old. As to the gender, the disease is more common in adult and elderly women. Below the age of 50 years, the incidence is similar in both genders^13^.

Of the 30 patients examined in this study, 18 (60%) were older than 60 years and 12 (40%) were <60 years old, a result that was similar to the one seen in the studies carried out by Marcondes et al.^13^ and Sousa et al.^10^.

As to gender, 21 were females (70%) and 9 were males (30%). This result is in agreement with the study from Marcondes et al.^12^, in which the prevalence in women surpassed the one in men and showed that in individuals <50 years old the incidence was similar in both genders.

Most patients (93.3%) had type 2 diabetes; only 2 patients (6.7%) had type 1 diabetes. These results are similar to those found by Antunes et al.3, Costa et al.^14^, Melgaço^15^ and Ogunbodede et al.^16^. To Neville et al.^17^ the oral manifestations in diabetic patients are generally limited to patients with type 1 diabetes. In this study, of the 30 patients, 93.3% had type 2 diabetes and of those, 80% presented at least one oral lesion.

The time between onset of the disease and the research varied between 5 and 45 years, with the intervals of 5 to 10 and 11 to 20 the most frequent periods with 44.4% of the sample in each range. In three patients (11.1%) the disease started between 21 and 45 years ago. Three patients did not know how to inform for how long they had had the disease.

In regards of the oral health of the patients, a fair status was found in 27 patients (90%), 2 patients (6.7%) presented good oral health and 1 patient (3.3%) showed poor oral health status. The literature consulted did not have any data related to this evaluation factor which suggests the need of further studies to include such criteria and the influence on the diabetic patient.

Lingual varicosity was diagnosed in 11 patients, corresponding to approximately 36.6% of the total abnormalities. The literature studied does not have a factor that links this abnormality to diabetes. Supposedly, this abnormality is related to the fact that it is a frequent semiological finding in elderly patients. This abnormality may also be related to the circulatory abnormalities typical of diabetes. These assumptions need further studies to be confirmed or ruled out.

Of the 30 patients evaluated, only 1 reported dry mouth (xerostomia), which represents only 3.33% of the individuals studied. This finding does not match the results of other studies^9^, ^10^, ^16^, ^18^, ^19^ which showed that this abnormality was predominant in diabetic patients examined by them. Carvalho^20^, Jordá et al.^21^ and Tófoli et al.^5^ believe that this abnormality, even if present, is not one of the most prevalent ones.

Gingivitis was present in 10% of the patients in this study. These findings are similar to those of the literature studied^10^, ^11^, ^14^, ^15^, ^18^, ^21^. In this study, these aspects are cited only for observation purposes, since the periodontal probing was not carried out in the patients because the evaluation of the caries and periodontal disease were not objects of evaluation.

Tommasi^9^, Sousa et al.^10^, Yuli Muller and Yuraima^11^, Neville et al.^17^ observed a high frequency of Candida infections in patients with diabetes mellitus. Quirino, Birgman and Paula^19^ linked this high frequency of Candida albicans infections with hyposalivation. The results of this study corroborate this observation in the sense that the number of patients affected by this disease was high (27.02%). In this study, of the 30 patients evaluated, 10 presented candidiasis (all cases of erythematous type) related to the use of prosthesis by these patients.

Guggenheimer et al.^12^ and Cristante et al.^18^ reported a high prevalence of angular cheilitis associated with the presence of Candida. The results of this study are in agreement with this idea since only 2 (5.40%) of 30 patients examined presented angular cheilitis, thus corroborating with indexes found in the studies performed by Ogunbodede et al.^16^ and Carvalho^20^.

Of the 30 patients evaluated, 2 (5.40%) presented traumatic ulcers; this condition was also seen in the studies carried out by Guggenheimer et al.^12^ and Quirino, Birgman and Paula^19^. The literature consulted did not reveal any factors that could relate this abnormality with diabetes. Guggenheimer et al.^12^ and Quirino, Birgman and Paula^19^ link the advanced age and the poor conditions of total prostheses with the presence of this abnormality. The use of total prostheses by the 2 patients who presented this abnormality in this study corroborate the ideas of Guggenheimer et al.^12^ and Quirino, Birgman and Paula^19^.

Russoto^22^ reported that the asymptomatic increased volume of the parotid gland in diabetic patients is not uncommon. Neville et al.^17^ and Sousa et al.^10^ stated that diabetes sialadenitis can be seen in patients with both types of diabetes. The findings of this study differ from the literature studied since no case of asymptomatic increased volume of the parotid gland was seen.

Oral lichen planus was not seen in this research, although it was present in approximately 5% of patients with type 2 diabetes in the studies carried out by Petrou-Amerikanous et al.^23^.

Guggenheimer et al.^12^ and Yuli Muller and Yuraima^11^ noticed a high prevalence of patients with fissured tongue and linked this condition with xerostomia. However, Costa et al.^14^ did not report any patients with lingual abnormalities. In this study, only 1 (3.33%) of the 30 patients presents this condition.

According to the results obtained in this study and according to the literature, which presents conflicting data, we suggest the need of additional studies with broader samples and nationwide coverage in order to better confirm the link between diabetes mellitus and the oral abnormalities found in the Brazilian population, thus filling several of the gaps still existing in this matter. These future studies will help healthcare professionals such as the otorhinolaryngologist and the dental surgeon who work on the diagnosis and treatment of oral lesions in diabetic patients.

## CONCLUSIONS

Most diabetic patients presented at least one lesion or abnormality of the oral mucosa. The abnormalities found included lingual varicosity, erythematous candidiasis, angular cheilitis, traumatic ulcer, fissured tongue, gingival hyperplasia, mucocele, xerostomia, petechiae, hyperkeratosis and atrophy of lingual papillae. The most frequent abnormalities were lingual varicosity and erythematous candidiasis.
